# Study of Volatile Secondary Metabolites Present in *Piper carpunya* Leaves and in the Traditional Ecuadorian Beverage Guaviduca

**DOI:** 10.3390/plants10020338

**Published:** 2021-02-10

**Authors:** Eduardo Valarezo, Jonathan Xavier Rivera, Edgar Coronel, Miguel Andrés Barzallo, James Calva, Luis Cartuche, Miguel Angel Meneses

**Affiliations:** Departamento de Química y Ciencias Exactas, Universidad Técnica Particular de Loja, Loja 110150, Ecuador; jxrivera2@utpl.edu.ec (J.X.R.); andres_edco@yahoo.com (E.C.); mabarzallo5@utpl.edu.ec (M.A.B.); jwcalva@utpl.edu.ec (J.C.); lecartuche@utpl.edu.ec (L.C.); mameneses@utpl.edu.ec (M.A.M.)

**Keywords:** guaviduca beverage, essential oil, *Piper carpunya*, 1,8-Cineole, enantioselective analysis, antibacterial activity, antioxidant activities, anticholinesterase activity

## Abstract

*Piper carpunya* Ruiz & Pav. is an aromatic shrub native to Ecuador, the leaves of which are used to prepare the traditional beverage Guaviduca. Different health benefits are attributed to the guaviduca beverage, which is consumed as a traditional and folk medicine. In this study, fresh *P. carpunya* leaves were collected in the winter and summer and subjected to hydrodistillation for the extraction of the essential oil. The guaviduca beverage was prepared by infusion in water and the volatile compounds were isolated by liquid–liquid extraction. Chemical composition and enantioselective analyses were performed by gas chromatography. The antibacterial activity was assayed against Gram-positive and Gram-negative bacteria. The scavenging radical properties of the essential oil was evaluated by 2,2-diphenyl-1-picrylhydryl (DPPH) and 2,2′-azinobis-3-ethylbenzothiazoline-6-sulfonic acid (ABTS) assays. The acetylcholinesterase inhibitory activity was measured using the spectrophotometric method. The chemical analysis allowed us to identify more than 98% of the compounds in all samples. The main constituent of the essential oil was 1,8-cineole (25.20 ± 1.31%) in *P. carpunya* collected in winter and (17.45 ± 2.33%) in *P. carpunya* collected in summer, while in the beverage, there was 14 mg/L. Safrole was identified in the essential oil (PCW 21.91 ± 2.79%; PCS 13.18 ± 1.72%) as well as in the beverage (2.43 ± 0.12 mg/L). Enantioselective analysis was used to investigate the enantiomeric ratio and excess of four chiral components. The essential oil presented a strong activity against *Klebsiella pneumoniae* with a MIC of 500 μg/mL and a very strong anticholinesterase activity with an IC_50_ of 36.42 ± 1.15 µg/mL.

## 1. Introduction

Guaviduca is a traditional beverage from the Ecuadorian Amazon prepared by the members of the Shuar community. The guaviduca beverage is made with leaves of the *Piper carpunya* species through infusion. *Piper carpunya* is a species belonging to the Piperaceae family. The Piperaceae family comprises about 2658 species belonging to 13 genera [1], of which the genus *Piper* is the largest, made up of around 1450 species [2]. Many of these are used as culinary spices and in traditional medicine as a result of their different biological properties [3]. In Ecuador, 450 species of the Piperaceae family have been registered, of which 212 belong to the genus *Piper*, with 157 native and 61 endemic varieties [4,5].

*Piper carpunya* Ruiz & Pav. (syn *Piper lenticellosum* C.D.C.) is an aromatic shrub native to Ecuador and Peru. It is widely distributed between 0–2000 m a.s.l. in the Andean and Amazon region [4], and it is even found in the Ecuadorian coastal region [6]. The plant is popularly known as “Guaviduca,” and the traditional preparations of the fresh or dried leaves in infusions or decoctions are used in folk medicine for their anti-inflammatory, antiulcer, antidiarrheal, and antiparasitic effects and wound healing properties (dermatological activity) [7,8]. The anti-inflammatory activity has been confirmed in mice models, such as in carrageenan-induced paw edema [9,10]. This plant has been shown to protect against gastric ulcers induced by non-steroidal anti-inflammatory drugs (NSAID) in rats [11]. In 2010, Quílez et al. concluded that the chemical compounds identified in *P. carpunya* are involved in the antisecretory, anti-inflammatory, and anti-*Helicobacter pylori* activities [12]. Previous phytochemical studies of *Piper carpunya* have reported the essential oil composition of the leaves and spikes [7,13]. These studies report compounds that may be harmful to human health. However, the presence of secondary metabolites in plant species changes due to variations in the ecological conditions, and genetic and environmental factors, the most important of these being temperature, moisture, soil, harvest period, geographical position, seasonal variations, and vegetative cycle [14]. The intrinsic and extrinsic factors are mainly related to the region where the species grow and the time of year in which the species are collected. The results obtained in previous studies support the use of *P. carpunya* in traditional medicine. However, there are no reports concerning the chemical compounds present in the guaviduca beverage prepared with the leaves of this species. This led us to investigate the chemical composition and enantiomeric distribution of the essential oil extracted from the *P. carpunya* species collected in winter and summer and also, the chemical composition from the volatile compounds present in this Ecuadorian beverage. Moreover, the antimicrobial, antioxidant, and anticholinesterase effects were assed for the volatile secondary metabolites present in *Piper carpunya* leaves.

## 2. Results

Through hydrodistillation in a Clevenger-type apparatus, we obtained ~40 mL of essential oil (EO) from 5000 g of *Piper carpunya*, which represents a yield of 0.81 ± 0.03% (*v*/*w*) (a mean of nine samples: three distillations x three collections) or 8.1 mL/Kg, which is considered as an intermediate yield [15], in reference to fresh plant material (68 ± 4% of moisture). The essential oil was a viscous liquid of subjective greenish-yellow color (RGB values of R:154, G:205, B:50 and CMYK values of C:0.25, M:0, Y:0.76, K:0.2). The essential oil’s relative density was *d*^20^ = 0.9321 ± 0.0039, its refractive index was *n*^20^ = 1.4877 ± 0.0010, and the specific rotation was [α]_D_^20^ = 3.5 ± 0.1° (dextro-rotatory).

### 2.1. Chemical Composition

The identification of compounds present in the *Piper carpunya* essential oil collected in the Ecuadorian Amazon and in the guaviduca beverage was carried out using mass spectral (MS) and retention index (RI) data. The results are summarized in Table 1. Considering that the guaviduca beverage is prepared and consumed throughout the year, the essential oil of the species collected in winter and summer was studied. The collections were performed every 15 days during two months for each season, in that way the variation in chemical composition was the greatest possible.

Thirty-four individual compounds were detected in the EO of *P. carpunya* collected in winter (PCW) and thirty compounds in the EO of the species collected in summer (PCS), which represent 98.86% and 98.26% of the total essential oil, respectively. Monoterpenes (hydrocarbons and oxygenates) were the most representative group with 83.38% in the PCW and 95.76% in PCS essential oil. In the PCW EO, eleven out of thirty-four compounds were oxygenated monoterpenes (OXM), with a contribution of 59.94%, nine were aliphatic monoterpene hydrocarbons (ALM), which represent 23,45% of the total, sesquiterpenes (hydrocarbons and oxygenates) represent about 14%, and it was not possible to determine the presence of aromatic monoterpene hydrocarbons (ARM). In the PCS EO, ALM compounds represent 55.58% and the OXM compounds 35.45%. Furthermore, a low amount of aliphatic sesquiterpene hydrocarbons (ALS) (1.93%) and traces of oxygenated sesquiterpene (OXS) were identified.

The main constituent of the essential oil was 1,8-cineole (CAS 470-82-6) 25.20 ± 1.31% in PCW and 17.45 ± 2.33% in PCS. Safrole (CAS 94-59-7) was the second compound with the highest percentage in the PCW oil with 21.91 ± 2.79%, and the third in the PCS oil with 13.18 ± 1.72%. The other main compounds were germacrene D (6.67 ± 0.44), α-terpinene (5.90 ± 0.44), and (E)-β-ocimene (5.66 ± 0.53) in the PCW oil, and (E)-β-ocimene (15.50 ± 2.53), (Z)-β-ocimene (9.45 ± 1.74), and α-Terpinene (8.93 ± 1.29) in the PCS EO. In the guaviduca beverage, compounds of the same chemical nature (monoterpenes and sesquiterpenes) as in the PCW and PCS essential oil samples were determined. The compounds with the highest abundance were 1,8-cineole with a concentration of around 14 mg/L, (E)-β-ocimene at 2.67 ± 0.13 mg/L, and safrole at 2.43 ± 0.12 mg/L.

### 2.2. Enantioselective Analysis

The enantioselective analysis was performed using 2,3-diethyl-6-tert-butyldimethylsilyl-β-cyclodextrin as a chiral selector [16,17]. Sabinene, phellandrene, linalool, and α-terpineol enantiomeric pairs were identified. The respective enantiomeric distribution and enantiomeric excess (e.e.) is presented in Table 2. In *Piper carpunya* EO, (1*R*, 5*R*)-(+)-Sabinene is the enantiomer with the highest enantiomeric distribution (93.3%) and an enantiomeric excess of 86.5%. The enantiomeric composition of essential oil can be used as a powerful tool for detecting adulterations [18]. These results further confirm that chiral secondary metabolites are often present in plants as enantiomeric mixtures.

### 2.3. Antimicrobial Activity

Considering that the compounds present in the essential oil exert synergistic or antagonistic effects, which produces a net activity [19], the antibacterial and antioxidant activities were determined as an estimate of their biological activity. The microdilution broth method was used to determine the minimum inhibitory concentration (MIC) against bacteria of the essential oils obtained from leaves of *P. carpunya*. The antimicrobial activity values are shown in Table 3. 

### 2.4. Antioxidant Capacity

*P. carpunya* essential oil was assessed for its antioxidant capacity using DPPH and ABTS radical scavenging assays. The antioxidant capacity was expressed as SC_50_, as a measure of the scavenging potential of the essential oil. The use of butylated hydroxytoluene (BHT) and Trolox as positive control allowed us to compare this value (Table 4). 

**Figure 1:**
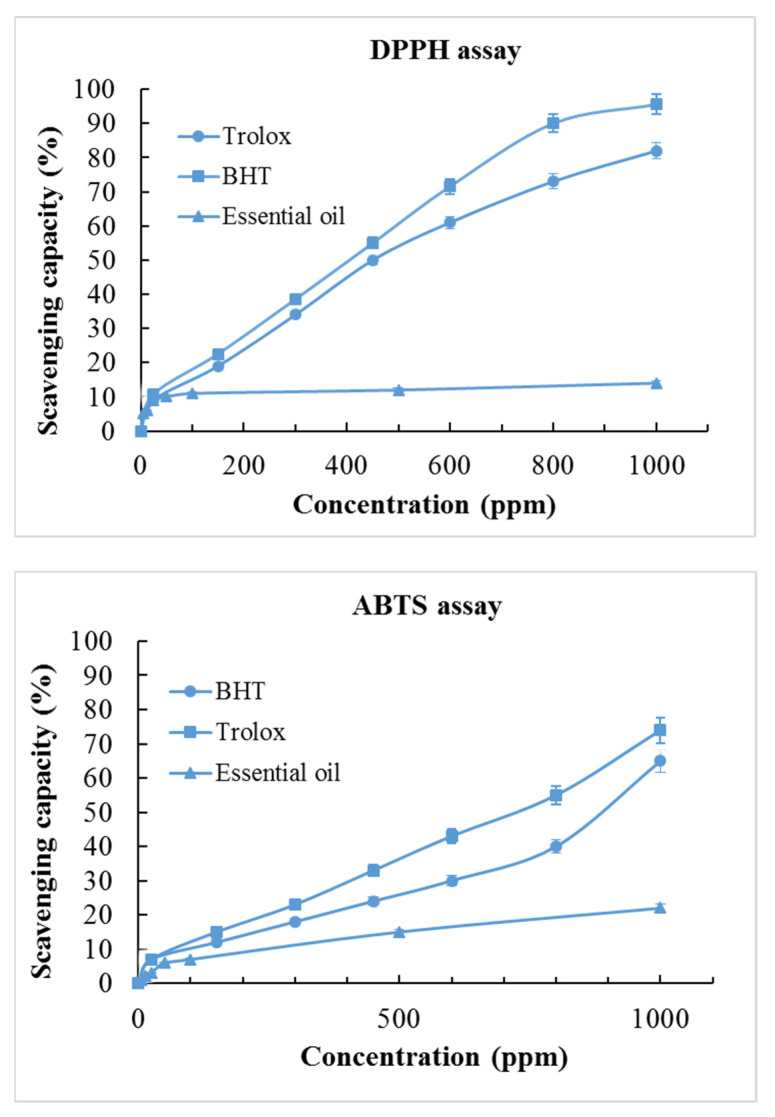
Antioxidant activity of *Piper carpunya* essential oil.

Through the DPPH method, the essential oils studied showed little antioxidant activity (12% of radical scavenge to 1000 ppm), and it was not possible to determine the SC_50_ values (>1000 μg/mL) at those concentrations. The SC_50_ for BHT and Trolox was close, but BHT (SC_50_ = 440 ± 30 μg/mL) was the most efficient. Employing the ABTS technique, an inhibition of 22% was obtained with 1000 ppm; however, the IC_50_ not was reached at the concentration ranges tested (Figure 1).

### 2.5. Anticholinesterase Activity

**Figure 2:**
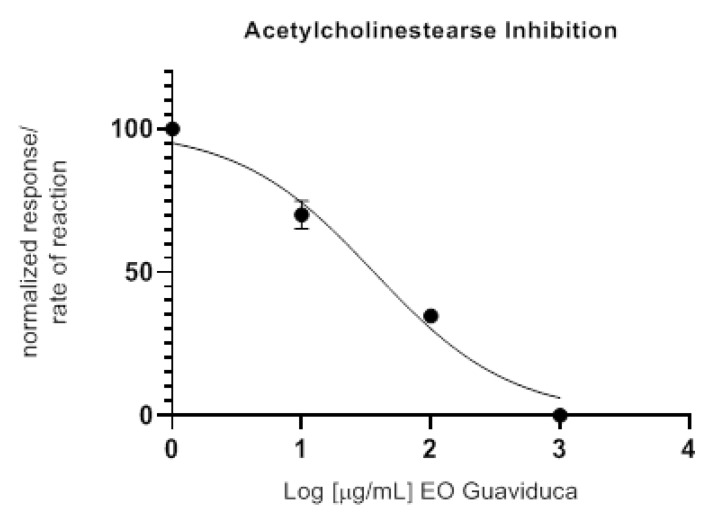
In vitro anticholinesterase activity of *Piper carpunya* essential oil expressed as log (concentration essential oil (EO)) vs. normalized response rate of reaction to calculate IC_50_ value.

The *P. carpunya* essential oil was assessed for its anticholinesterase potential by measuring the rate of reaction of AChE against three different concentrations of the essential oil (Figure 2). The IC_50_ value obtained for guaviduca EO was 36.42 ± 1.15 µg/mL, which can be considered to be very strong as compared with the related EO from *Piper* spp., such as *P*. *betle*, *P*. *asutrosinense*, *P*. *puberulum*, *P. flaviflorum*, and *P hispidimervium*, in which the inhibition of AChE ranged from 12.4 mg/mL to 1.5 mg/mL as reported by Xiang et al. [20]. Donepezil exhibited an IC_50_ value of 13.80 ± 1.01 nM. In traditional Ayurvedic and Chinese medicine practices, plants have been extensively used for cognitive disorder treatments, including Alzheimer’s disease (AD), a neurodegenerative disorder characterized mainly by impaired memory and behavior. According to the cholinergic hypothesis, memory impairment in patients suffering AD results from a deficit in cholinergic function in the brain [21]. Thus, the most relevant and successful approach was the inhibition of Cholinesterases to increase the levels of Acetylcholine. To date, only tacrine, donepezil, rivastigmine, and galanthamine have been approved by the FDA for AD symptomatic treatment [22] and only DON (donezepil), RIV (rivastigmine), and GAL (galantamine) are strictly known as cholinesterase inhibitors.

## 3. Discussion

Various compounds identified in the present investigation, such as 1,8-cineole (13.0%), safrole (14.9%), and spatulenol (9.8%), were also identified as major compounds in a study carried out on essential oil from leaves of *P. carpunya* species grown in the Peruvian Amazon [13] (the stage is not specified), although, with different percentages. In 2019, Ballesteros et al. determined that *P. carpunya* essential oil collected in the balsamic period (before flowering) in the Kutuku Experimental Station, Morona Santiago province, Ecuador, is mainly composed of piperitone (26.2%). According to reports, this oil only contains 2.2% of safrole and 4% of 1,8-cineole [7]. Piperitone (33.97%), 1,8 cineole (11.92%), limonene (11.07%), and safrole (8.18%) were identified as major constituents of an essential oil of this species collected in July (the stage is not specified) in Guayas, an Ecuadorian coastal province [6]. The chemical composition of the essential oils of the same species varies in terms of intrinsic and extrinsic factors [14]. For this reason, the chemical composition of the compounds in the *P. carpunya* leaves was determined as the source of the compounds present in the beverage.

The compounds present in the essential oil, such as 1,8-cineole, β-ocimene (E and Z), safrole, and others (Table 1), are also present, in different concentrations, in the guaviduca beverage. However, the composition of the beverage is more closely related to the composition of the essential oil extracted from the leaves collected in the summer, which shows that the compounds present in the beverage are similar qualitatively to those in the leaves used to make it. In the case of the guaviduca beverage, which is consumed mainly for its anti-inflammatory properties, 1,8-cineole (eucalyptol) is present, which, due to its anti-inflammatory, antimicrobial, broncholytic, and mucolytic properties, is used to treat diseases of the respiratory tract [23]. In 2004, Santos [24] stated that 1,8-cineole caused repletion of glutathione and reduced myeloperoxidase activity, which causes reduction of colon inflammation in rats, confirming that 1,8-cineole has an anti-inflammatory action and suggesting its potential use in the prevention of ulceration and gastrointestinal inflammation as a dietary flavoring agent. 1,8-Cineole produced a notable increase in glucuronyltransferase activity when it was given via food/water to rats for 4 days or by aerosol for 5–8 days in doses of 500 mg/kg/day or 1000 mg/kg/day. This increase returned to normal after 20 days [19]. This effect was also studied by Yokota et al. in 1988 [25]. In their study, Yokota et al. established that eugenol dose-dependently increased levels of hepatic UDP-glucuronyltransferase when given in the diet at 1%, 3%, or 5% for 23 days. The antiulcer, antidiarrheal, and antiparasitic capacity associated with the guaviduca beverage may be due to the presence of β-ocimene (E and Z), which is associated with EOs with anticonvulsant activity, antifungal activity, antitumor activity, and pest resistance [26,27,28,29]

In *P. carpunya* EO and in the beverage prepared with this species, compounds with medicinal properties, such as those mentioned above, are present; however, compounds that are harmful to human health, such as safrole, are also present. In scientific reports, safrole is considered a human carcinogen based on sufficient evidence of hepatocarcinogenic in rats and mice. The US EPA (United States Environmental Protection Agency) has evaluated the carcinogenicity of safrole and classifies it as possibly carcinogenic to humans (Group B2: the agent (mixture) is possibly carcinogenic to humans). The major toxicities of safrole and isosafrole come from their carcinogenic nature after the oxidation of the allyl side chain and the oxidation of the methylenedioxy group [30]. Safrole is oxidized into 1-hydroxysafrole, isosafrole, and dihydrosafrole, which are all carcinogenic (Bogusz, 2008) and have been found to modify DNA covalently [31]. The effects of safrole can differ due to the exposure conditions, e.g., short-term vs. chronic toxicity [30]. Safrole cannot be used as fragrance ingredient: the International Fragrance Association (IFRA) recommends that the essential oils containing safrole should not be used at levels in which the total concentration exceeds 0.01% in consumer products [19,32]. The use of natural substances containing safrole is regulated by legal restriction, e.g., in Europe, a maximum concentration of 1 ppm in food and beverages is allowed [33]; in the USA, it has been prohibited in soft drinks in since 1970s; it is allowed in China (State Food and Drug Administration of China, SFDA of China) in concentrations below 1 mg/L [34].

Regarding the in vitro antimicrobial activity of natural products, in 2017, Van Vuuren and Holl suggested a classification scale for extracts and essential oil [35]. In summary, for essential oils, the activity is classified as very strong when MIC is ≤100 μg/mL, strong when MIC is between 101 and 500 μg/mL, and moderate for values of MIC between 500 to 1000 μg/mL. On the basis of these criteria, the essential oil from *P. carpunya* presented a strong activity against Gram-negative bacterium *Klebsiella pneumoniae* (ATCC 9997) with a MIC of 500 μg/mL. However, according to Cos et al., to correct for the many false positives, for all anti-infective bioassays, the IC_50_ valid values should be lower than 100 μg/mL for mixtures of compounds and lower than 25 μM in the case of pure compounds [36]. Additionally, the essential oil was tested against two Gram-positive bacteria, but the EO did not show activity at the maximum concentration tested (1000 μg/mL). The loss of activity is frequently caused for the phenomenon of additive or synergistic effects in mixtures or extracts [36].

The antibacterial capacity against *K. pneumoniae* of the *P. carpunya* EO may be due to its high concentration in 1,8-cineole, which has been reported as a compound with antibacterial capacity [37]. Furthermore, 1,8-cineole showed significant levels of synergistic interaction when combined with antimicrobial compounds such as chlorhexidine gluconate (CHG) against *Staphylococcus aureus*, *S. aureus*, *Escherichia coli*, *Klebsiella pneumoniae*, *Enterococcus faecalis*, and *Candida albicans* [38]. On the other hand, it has been determined that essential oils with a high safrole content (75–85%) such as that extracted from *Piper xylosteoides* [39] and that from *Piper hispidinervum* [40,41] present weak activity against Gram-positive and Gram-negative bacteria. Azuero, et al. [42] evaluated the antimicrobial effect of methanolic extracts of *P. carpunya* leaves and determined a high antibacterial effect against *E. coli* and a weak effect against *P. aeruginosa*; however, no effect against *S. aureus* was determined. In the same study, *P*. *carpunya* showed a high fungicidal action against *C. albicans*. Quílez et al. [12] isolated different compounds from an ethanolic extract, and the identified compounds were reported to present antibacterial activity [43,44,45].

The absence of antioxidant activity in the DPPH assay, for the essential oil in this study, may be explained by the fact that terpene compounds are not capable of donating a hydrogen atom [46]. However, in the investigations of antioxidant activity of lipophilic substances, the ABTS method has been proven to be appropriate for essential oils [47]. The results of this study indicate that the activity of compounds with proven in vivo antioxidant activity, such as 1,8-cineole [48], are being antagonized by other compounds present in the essential oil, since the EO did not present antioxidant activity in the in vitro test, which is an assay directly related to the nature of the compound (the compound must not be metabolized) [49] and of which the mechanism is known [50]. We suggest that the absence of in vitro antioxidant activity in *P. carpunya* essential oils mainly consisting of monoterpene and sesquiterpene, which have antioxidant activity, may be due to antagonism with other compounds. 

On the other hand, in vitro and in vivo assays showed that the *P. carpunya* extract affects the action of neutrophils, reducing the release of myeloperoxidase enzymes which are involved in the pro-inflammatory activity. In addition, *P. carpunya* extract was involved in the preservation of antioxidant enzyme activity in mucosa exposed to damage [11]. Quílez et al. reported chemical compounds such as dihydrochalcones, isovitexin, and vitexin, isolated from *P. carpunya*, which have potent antioxidant free radical scavenging activities. Antioxidant activity is one of the proposed mechanisms of action for establishing anti-inflammatory properties [12]. Ballesteros et al. reported a weak radical scavenging ability (IC_50_ 159.80 ± 3.4 μg/mL DPPH assay) of the *P. carpunya* essential oil. The IC_50_ value of pure compounds was higher, indicating that this activity could correspond to the mixture with the minor compounds. Thus, it changes with the composition of the essential oil [7].

## 4. Materials and Methods

### 4.1. Materials

Dichloromethane, sodium sulfate anhydrous, 2,2-diphenyl-1-picrylhydryl (DPPH), 2,2′-azinobis-3-ethylbenzothiazoline-6-sulfonic acid (ABTS), butylated hydroxytoluene (BHT), acetylthiocholine (AcSCh, SIGMA-O1480), phosphate buffered saline (PBS, SIGMA-P4417), 5,5′-Dithiobis(2-nitrobenzoic acid) (DTNB, SIGMA-D218200), acetylcholinesterase (AChE, SIGMA-C3389), donepezil (SIGMA-D6821), methanol (MeOH), tris hydrochloride (Tris-HCl), and magnesium chloride hexahydrate were purchased from Sigma-Aldrich. Aliphatic hydrocarbons standard was purchased from CHEM SERVICE under the name Diesel Range Organics Mixture #2-GRO/DRO, with the code M-TPH6X4-1ML. Helium was purchased from INDURA Ecuador. Microbiological media as Sabourad and Mueller Himton Broth were purchased from DIPCO. Dimethylsulfoxide (DMSO) was obtained from Fisher. All chemicals were of analytical grade and used without further purifications.

### 4.2. Plant Material

**Figure 3:**
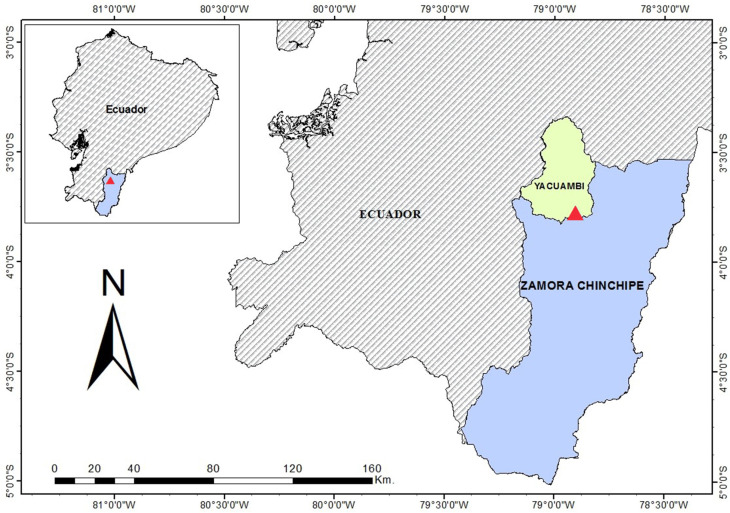
Collection sector of the *Piper carpunya* species in the Ecuadorian Amazon.

The collection of the species was performed with the authorization (N° 001-IC-FLO-DBAP-VS-DRLZCH-MA) of the Ministerio del Ambiente de Ecuador (MAE), in the surroundings of the Shuar community “El Kiim” of the La Paz parish, Yacuambi canton, Zamora Chinchipe province (Ecuadorian Amazon), at a latitude of 3°47′06″ S and a longitude of 78°54′16″ W (Figure 3). The collection was performed in the months of February–March (in winter; Ecuador only has two seasons) and October–November (summer) at an altitude of 1025 m a.s.l. The samples collected in winter were in the fruiting stage and were identified as PCW; the samples collected in the summer in the foliation stage were designated as PCS. Airtight plastic containers were used for storage and transfer of the plant material until its use. The collection and transfer temperature was 20–24 °C (room temperature), and the pressure was around 90 KPa (room pressure). 

The botanical identification of specimens was performed at the herbarium of the “Universidad Nacional de Loja” by Dr. Bolivar Merino. A voucher specimen number PPN-pi-010 is preserved in the Herbarium of the Universidad Técnica Particular de Loja. The method AOAC 930.04-1930, Loss on drying (Moisture) in plants, was used to determine the moisture of plant material.

### 4.3. Essential Oil Isolation

The material was processed fresh, immediately after arriving at the laboratory, between 2 h and 4 h after being collected. The plant material was submitted to hydrodistillation for 4 h in a Clevenger-type apparatus. Subsequently, moisture was removed from the collected essential oil by the addition of anhydrous sodium sulphate, and finally, it was stored in amber-sealed vials at 4 °C to protect it from light until being used in the subsequent analysis [51].

### 4.4. Determination of Physical Properties of Essential Oil

Three physical properties were determined for the essential oil: relative density, refractive index, and optical rotation. The standard AFNOR NF T 75-111 [52] (equivalent to the standard ISO 279:1998) was used to determine the relative density, the standard AFNOR NF T 75-112 [52] (similar to ISO 279:1998) for the refractive index, and the standard method ISO 592:1998 for optical rotation. For relative density, an analytical balance (model Mettler AC 100, ±0.0001) and a pycnometer (1 mL) were used; for refraction index, a refractometer (model ABBE) was used; and for optical rotation, an automatic polarimeter (model Mrc-P810) was used. Measurements were performed at 20 °C and repeated three times.

### 4.5. Elaboration of the Beverage

The preparation of the guaviduca beverage was carried out according to the indications of the members of the El Kiim community. We placed 32 g of fresh guaviduca leaves (between two and three leaves of PCS) into 1 L of boiling water. The cooking process was immediately stopped (infusion), then the leaves were mixed with the water, and allowed to cool in a closed container. The container was periodically shaken lightly. The infusion rested until it reached room temperature (20 °C) and was then filtered through a sieve mesh (850 μm) (No. 20 ASTM E11). The liquid fraction was stored in airtight amber containers at 4 °C (refrigeration) until being used in the subsequent analysis.

### 4.6. Extraction of Compounds from Beverage

For the isolation of the compounds, 300 mL of the beverage was placed in a settling funnel, and 4.5 mL of dichloromethane (1.5% *v*/*v* solution) was added. The solution was gently shaken for one full minute every 10 min. After 1 h, the non-aqueous fraction was decanted, collected in amber vials, and refrigerated until being used in the subsequent analysis.

### 4.7. Compounds Identification

The quantitative and qualitative identification of the volatile secondary metabolites present in *Piper carpunya* leaves and guaviduca beverage was carried out using gas chromatography (GC). Quantitative analyses were carry out using an Agilent gas chromatograph (model 6890N series) equipped with a flame ionization detector (GC/FID) according to the procedure described by Valarezo et al. [53]. The relative amounts of individual components were calculated based on the GC peak area (FID response) without using a correction factor. For quantitative analysis, an external calibration curve was used. The calibration curve was obtained by injecting six dilutions of limonene. The dilutions were obtained by diluting 0.1, 0.25, 0.5, 1, 5, and 10 mg of limonene to 10 mL with dichloromethane. The calibration curve achieved a R^2^ > 0.99. Qualitative analyses were performed using an Agilent gas chromatograph (model 6890N series, Agilent Technologies, Santa Clara, CA, USA) coupled to a mass spectrometer (quadrupole) detector (model Agilent series 5973 inert) (GC/MS) (Agilent Technologies, Santa Clara, CA, USA) as per the procedures described earlier by Valarezo et al. [53]. Identification of the constituents was carried out by comparing the retention index (RI) and mass spectral data (MS) with those in the literature [54,55,56]. For qualitative and quantitative analyses, an automatic injector (Agilent 7683 automatic liquid sampler, Agilent Technologies, Santa Clara, CA, USA) and a nonpolar column (J&W DB-5ms Ultra Inert GC column: 30 m × 0.25 mm × 0.25 μm; Agilent Technologies, Santa Clara, CA, USA) were used.

### 4.8. Enantioselective Analysis

For enantioselective analysis, an Agilent gas chromatograph (model 6890N series) was used coupled to a mass spectrometer detector (quadrupole model Agilent series 5973 inert) using an enantioselective column with a stationary phase (2,3-diethyl-6-tert-butyldimethylsilyl-β-cyclodextrin). The chromatographic run was performed with a temperature ramp of 2 °C/min from 50 °C (mainatined for 2 min) to 220 °C (mainatined for 2 min).

### 4.9. Antimicrobial Activity

Antimicrobial activity was determined against five Gram-negative bacteria (*Pseudomonas aeruginosa* (ATCC 27853), *Klebsiella pneumoniae* (ATCC 9997), *Proteus vulgaris* (ATCC 8427), *Escherichia coli* (ATCC 25922), *Salmonella typhimurium* (LT2)) and two Gram-positive bacteria (*Enterococcus faecalis* (ATCC 29212) and *Staphylococcus aureus* (ATCC 25923)), according to the procedure previously described by Valarezo et al. [57]. The bacterial strains were incubated in Müeller–Hinton (MH) broth and DMSO was used to dissolve the essential oil. Gentamicine was used as a positive control for *P. aeruginosa*, *K. pneumonia*, *P. vulgaris*, *E. coli*, *S. aureus*, and ampicilline for *E. faecalis* and *S. typhimurium*. DMSO was used as a negative control and the results are reported as minimum inhibitory concentration (MIC) (the lowest concentration of sample capable of inhibiting all visible signs of growth of the microorganism).

### 4.10. Antioxidant Capacity

#### 4.10.1. DPPH Radical Scavenging Activity

The DPPH free radical scavenging activity of oils was measured based on the scavenging activity of the stabilized 2,2-diphenyl-1-picrylhydryl radical. 

The procedure was performed according to Brand Williams et al. [58] and Thaipong et al. [59]. The method consists in the preparation of a stable solution of DPPH with methanol until the absorbance is adjusted to 1.1 ± 0.02 units at a wavelength of 515 nm in a UV spectrophotometer (Genesys 10S UV.Vis Spectrophotometer, Thermo Fisher Scientific, Waltham, MA, USA). For the antiradical evaluation, 2850 µL of DPPH solution were allowed to react with 150 µL of *P. carpunya* essential oil at different concentrations, at room temperature and protected from light for 24 h. After that, the final absorbance was measured at the same wavelength. The same procedure was followed for positive controls BHT and Trolox, while methanol was used as a blank control. 

The free radical-scavenging capacity was expressed as percentage and was calculated by: R (%) = [(As − Ai)/As] * 100%, where Ai represents the final absorbance of the DPPH after reacting with essential oil or positive controls, and As is the absorbance of the DPPH reaction with the blank control. The plotting of R (%) against the concentration of the essential oil allowed us to identify the oil concentration that provided 50% scavenging effect (SC_50_) of DPPH free radicals. All measurements were performed in triplicate and reported as the average value.

#### 4.10.2. ABTS Radical Cation Scavenging Activity

The ABTS antioxidant method was performed according the modifications of Thaipong et al. [59] to the initial procedure described by Arnao et al. [60]. Briefly, the ABTS radical cation (ABTS•+) was produced after the reaction for 12 h of equal volumes of two stock solutions (7.4 µM ABTS and 2.6 µM potassium persulfate). After that, an ABTS standard solution was prepared by dissolution in methanol until reaching an absorbance of 1.1 ± 0.02, at a wavelength of 734 nm. The antiradical evaluation was evaluated when 2850 µL of the ABTS standard solution was allowed to react with 150 µL of the *P. carpunya* essential oil at different concentrations, at room temperature, and protected from light for 2 h. Then, the final absorbance was registered. Similar to the DPPH analysis, BHT and Trolox were used as positive controls, while deionized water was used as blank control. ABTS radical scavenging activity was calculated by: SA (%) = (Ao − Ai)/Ao * 100%, where Ao represents the absorbance of the ABTS•+ reaction with the blank control, and Ai is the final absorbance of ABTS•+ after reacting with the essential oil or positive controls. The SC_50_ was determined by comparing the graph of SA (%) against the concentration of essential oil. Every analysis was performed in triplicate and the average value was reported.

### 4.11. Anticholinesterase Activity

The AChE inhibitory activity was measured using the spectrophotometric method developed by Ellman, et al. [61], with slight modifications as suggested by Rhee, et al. [62]. Acetylthiocholine was used as the substrate to detect the inhibition of AChE. The reaction mixture contained 40 µL of Buffer C (Tris–HCl, 50 mM, pH 8, containing 0.1 M of sodium chloride and 0.02 M of magnesium chloride hexahydrate), 20 μL of the tested sample solution, 20 µL of AcSCh (15 mM, PBS pH 7.4), and 100 µL of DTNB (3 Mm, dissolved in Buffer C). The mixture was pre-incubated for 3 min at 25 °C. Finally, the enzymatic reaction was started with the addition of 20 µL of 0.5 U/mL AChE (137 U/mg solid) and then incubated at 25 °C for 30 min. The amount of product released was monitored in an EPOCH 2 (BIOTEK ^®^) microplate reader every 1 min at 405 nm. All reactions were performed in triplicate in a 96-well microplate. Tested sample solutions from the essential oil were made by dissolving 10 mg in 1 mL MeOH. Two more dilutions were included in MEOH (10× dilution) to obtain final concentrations (1000, 100, and 10 ug/mL) of essential oil in MeOH. The IC_50_ value was calculated by curve fitting of data (non-linear regression analysis, PRISM 8.0.1, GraphPad, San Diego, CA, USA). Donepezil was used as positive control. Any increase in absorbance because of spontaneous hydrolysis of the substrate was corrected by subtracting the absorbance at the end of pre-incubation from the absorbance measured after the addition of the enzyme. The IC_50_ value was measured from the corresponding rate of the reaction curve with Graph Pad v8.0.1.5.

## 5. Conclusions

A complete characterization of the chemical composition, enantioselective analysis, physical properties, and antimicrobial, antioxidant, and AChE inhibitor activities was conducted for *Piper* *carpunya* essential oil. The results show that the main compounds are present in the essential oil whether it is made from leaves collected in winter or summer. The compounds identified in the guaviduca beverage were volatile secondary metabolites, which are of the same nature as those present in the essential oil. A total of 34 compounds were identified in the EO collected in winter and thirty compounds in the EO collected in summer, while in the beverage there were twenty-three volatile compounds. The chemical compounds found in the PCW and PCS were mainly grouped into oxygenated monoterpenes and aliphatic monoterpene hydrocarbons. The presence of safrole in the beverage was lower than in the essential oil, with a concentration of 2.43 ± 0.12 mg/L; however, this level is higher than the recommendations in Europe and China (a maximum of 1 ppm). This study of the chemical volatile compounds present in *P. carpunya* and in the guaviduca beverage contributes to our knowledge concerning the use traditional of this species. Despite the fact that guaviduca beverage contains beneficial compounds such as 1,8-cineole and β-ocimene, also contains safrole, which is a compound that is harmful to health. It was determined that safrole is present in beverage in an amount not acceptable for the international committees (US EPA, IFRA, SFDA of China, etc.). Further studies should be conducted to determine the antimicrobial and mainly, the cholinesterase inhibitory activity from the major compounds, to value the real potential of the essential oil from guaviduca despite the presence of safrole. Letal acute toxicity in mice can be proposed for a further study.

## Figures and Tables

**Table 1:** Chemical composition of volatile compounds from *Piper carpunya* leaves collected in winter (PCW) and summer (PCS) and from the guaviduca beverage.

Peak	Compound	RI	RI^ref^	PCW		PCS		Beverage		Type	CF	MM
				% ^a^	SD	% ^a^	SD	mg/L	SD			(Da)
1	α-Thujene	926	924	-	-	0.16	0.05	0.21	0.01	ALM	C_10_H_16_	136.13
2	α-Pinene	932	932	2.65	0.34	4.71	0.44	0.79	0.04	ALM	C_10_H_16_	136.13
3	Sabinene	969	969	-	-	1.55	0.04	0.61	0.03	ALM	C_10_H_16_	136.13
4	β-Pinene	973	974	2.39	0.32	3.63	0.01	0.55	0.03	ALM	C_10_H_16_	136.13
5	Myrcene	986	988	0.70	0.09	1.62	0.16	0.61	0.03	ALM	C_10_H_16_	136.13
6	δ-2-Carene	1004	1001	0.17	0.04	0.14	0.01	-	-	ALM	C_10_H_16_	136.13
7	α-Terpinene	1013	1014	5.90	0.44	8.93	1.29	1.83	0.09	ALM	C_10_H_16_	136.13
8	ρ-Cymene	1021	1020	-	-	4.72	0.41	1.23	0.06	ARM	C_10_H_14_	134.10
9	β-Phellandrene	1026	1025	-	-	4.63	1.20	-	-	ALM	C_10_H_16_	136.13
10	1.8-Cineole	1029	1026	25.20	1.31	17.45	2.33	14.41	0.72	OXM	C_10_H_18_O	154.14
11	(Z)-β-Ocimene	1034	1032	3.40	0.32	9.45	1.74	1.77	0.09	ALM	C_10_H_16_	136.13
12	(E)-β-Ocimene	1045	1044	5.66	0.53	15.50	2.53	2.67	0.13	ALM	C_10_H_16_	136.13
13	γ-Terpinene	1055	1054	2.43	0.20	5.18	1.27	0.65	0.03	ALM	C_10_H_16_	136.13
14	cis-Sabinene hydrate	1063	1065	0.34	0.02	-	-	-	-	OXM	C_10_H_18_O	154.14
15	Terpinolene	1082	1086	0.14	0.02	0.09	0.01	-	-	ALM	C_10_H_16_	136.13
16	Linalool	1099	1095	1.06	0.05	0.44	0.05	-	-	OXM	C_10_H_18_O	154.14
17	Chrysanthenone	1121	1124	1.35	0.05	-	-	-	-	OXM	C_10_H_14_O	150.10
18	Camphor	1140	1141	0.15	0.01	-	-	-	-	OXM	C_10_H_16_O	152.12
19	Terpinen-4-ol	1170	1174	1.60	0.22	0.17	0.01	0.86	0.04	OXM	C_10_H_18_O	154.14
20	α-Terpineol	1195	1186	3.15	0.16	1.36	0.41	0.15	0.01	OXM	C_10_H_18_O	154.13
21	Ascaridole	1239	1234	2.35	0.14	1.04	0.32	0.88	0.04	OXM	C_10_H_16_O_2_	168.12
22	Cumin aldehyde	1242	1238	-	-	0.18	0.06	-	-	OXM	C_10_H_12_O	148.09
23	Carvone	1244	1239	-	-	0.43	0.07	>0.1	-	OXM	C_10_H_14_O	150.10
24	Piperitone	1248	1249	0.26	0.03	-	-	-	-	OXM	C_10_H_16_O	152.12
25	α-Terpinen-7-al	1280	1283	0.09	0.03	-	-	-	-	OXM	C_10_H_14_O	150.10
26	Safrole	1291	1285	21.91	2.79	13.18	1.72	2.43	0.12	OXM	C_10_H_10_O_2_	162.07
27	Thymol	1294	1289	-	-	0.47	0.06	-	-	OXM	C_10_H_14_O	150.10
28	Carvacrol	1301	1298	-	-	0.26	0.04	0.79	0.04	OXM	C_10_H_14_O	150.10
29	(Z)-Methyl cinnamate	1304	1299	2.48	0.02	0.48	0.34	0.59	0.03	OXM	C_10_H_10_O_2_	162.07
30	Thymol acetate	1346	1349	0.28	0.14	tr	-	-	-	OTC	C_12_H_16_O_2_	192.12
31	trans-Caryophyllene	1405	1417	0.62	0.27	-	-	-	-	ALS	C_15_H_24_	204.19
32	ρ-Cymen-7-ol acetate	1421	1421	1.45	0.13	0.57	0.04	0.14	0.01	OTC	C_12_H_16_O_2_	192.12
33	α-Humulene	1451	1452	0.25	0.10	-	-	-	-	ALS	C_15_H_24_	204.19
34	(E)-β-Farnesene	1459	1454	0.22	0.21	0.16	0.01	>0.1	-	ALS	C_15_H_24_	204.19
35	Germacrene D	1476	1480	6.67	0.44	0.92	0.23	0.21	0.01	ALS	C_15_H_24_	204.19
36	Bicyclogermacrene	1495	1500	3.58	0.23	0.85	0.41	>0.1	-	ALS	C_15_H_24_	204.19
37	(E. E)-α-Farnesene	1503	1505	0.38	0.03	-	-	-	-	ALS	C_15_H_24_	204.19
38	β-Sesquiphellandrene	1519	1521	0.12	0.01	-	-	-	-	ALS	C_15_H_24_	204.19
39	δ-Cadinene	1521	1522	0.27	0.02	-	-	-	-	ALS	C_15_H_24_	204.19
40	(E)-Nerolidol	1557	1561	tr	-	-	-	-	-	OXS	C_15_H_26_O	222.20
41	Spathulenol	1573	1577	1.18	0.10	tr	-	>0.1	-	OXS	C_15_H_24_O	220.18
42	Carotol	1590	1594	0.48	0.19	-	-	-	-	OXS	C_15_H_26_O	222.20
*Aliphatic monoterpene hydrocarbons (ALM)*				23.45		55.58						
*Aromatic monoterpene hydrocarbons (ARM)*				-		4.72						
*Oxygenated monoterpenes (OXM)*				59.94		35.45						
*Aliphatic sesquiterpene hydrocarbons (ALS)*				12.09		1.93						
*Oxygenated sesquiterpene (OXS)*				1.66		tr						
Other compounds (OTC)				1.72		0.57						
Total identified				98.86		98.26						

^a^ Mean of nine determinations; RI: calculated retention indices; RI^ref^: references retention indices; tr. trace (<0.05%); SD: standard deviation; -: not detected; CF: Chemical Formula; MM: Monoisotopic mass.

**Table 2:** Enantioselective analysis of various chiral constituents of essential oil from *Piper carpunya*.

Enantiomers	RT	RI	Enantiomeric Distribution	e.e.
	min	min	%	%
(1*R*,5*R*)-(+)-Sabinene	7.6	968	93.3	86.5
(1*S*,5*S*)-(-)-Sabinene	7.83	973	6.7	
(R)-(−)-α-Phellandrene	17.09	1019	78.3	56.6
(S)-(+)-α-Phellandrene	18.64	1026	21.7	
(*S*)-(+)-linalool	24.54	1107	76.6	53.1
(*R*)-(-)-Linalool	24.68	1109	23.4	
(*R*)-(+)-α-Terpineol	30.56	1187	70.0	40.0
(*S*)-(-)-α-Terpineol	30.74	1189	30.0	

RT, Retention time; e.e. = enantiomeric excess.

**Table 3:** Antibacterial activity of essential oil from *Piper carpunya*, given as minimal inhibitory concentration (MIC, μg/mL).

Microorganism	*P. carpunya*	Positive Control
	MIC (μg/mL) ^a^	
Gram-negative bacteria		
*Pseudomonas aeruginosa* (ATCC 27853)	>1000	15.6
*Klebsiella pneumoniae* (ATCC 9997)	500	1.95
*Proteus vulgaris* (ATCC 8427)	>1000	3.91
*Escherichia coli* (ATCC 25922)	>1000	1.95
*Salmonella typhimurium* (LT2)	>1000	1.95
Gram-positive bacteria		
*Enterococcus faecalis* (ATCC 29212)	>1000	7.81
*Staphylococcus aureus* (ATCC 25923)	>1000	7.81

^a^ Mean of nine determinations.

**Table 4:** Antioxidant activity of essential oils of *Piper carpunya.*

Sample	DPPH	ABTS
	SC_50_ * (μg/mL)	
Essential oil	>1000	>1000
BHT	440 ± 30	840 ± 30
Trolox	450 ± 50	650 ± 20

* IC_50_ = Inhibition Concentration of 50%.

## Data Availability

Not applicable.
